# Supplementary figures and images for: Clinical predictors for etiology of acute diarrhea in children in resource-limited settings
Source: PLoS Negl Trop Dis. 2020 Oct 9;14(10):e0008677. doi: 10.1371/journal.pntd.0008677 (PMC7588112; doi:10.1371/journal.pntd.0008677)

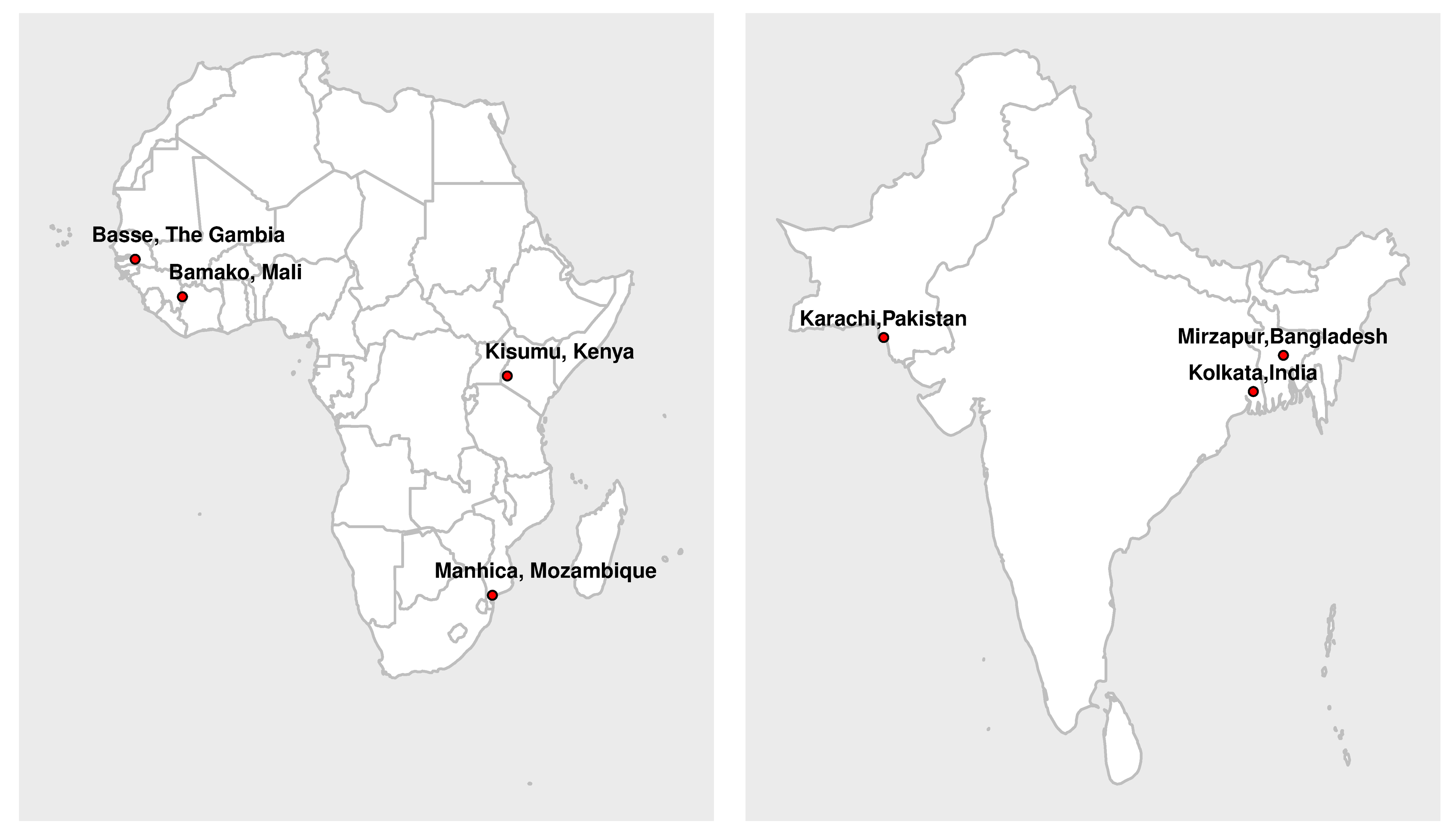

Supplement: S1 Fig — Right map shows the locations of 3 study sites in South Asia. The map was generated using the get_map and ggmap functions in R version 3.6.1. (TIF) [file pntd.0008677.s003.tif]

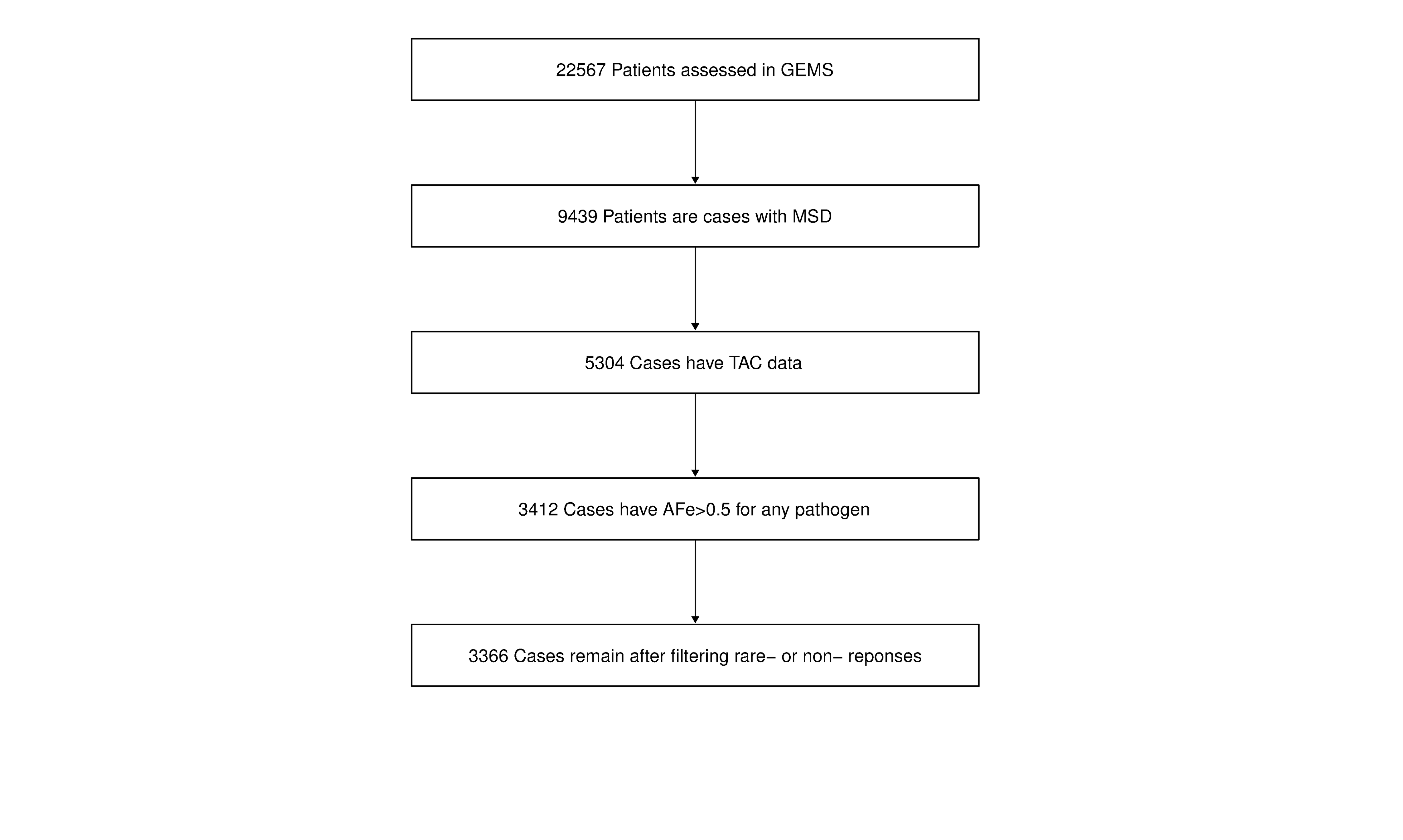

Supplement: S2 Fig — (TIF) [file pntd.0008677.s004.tif]

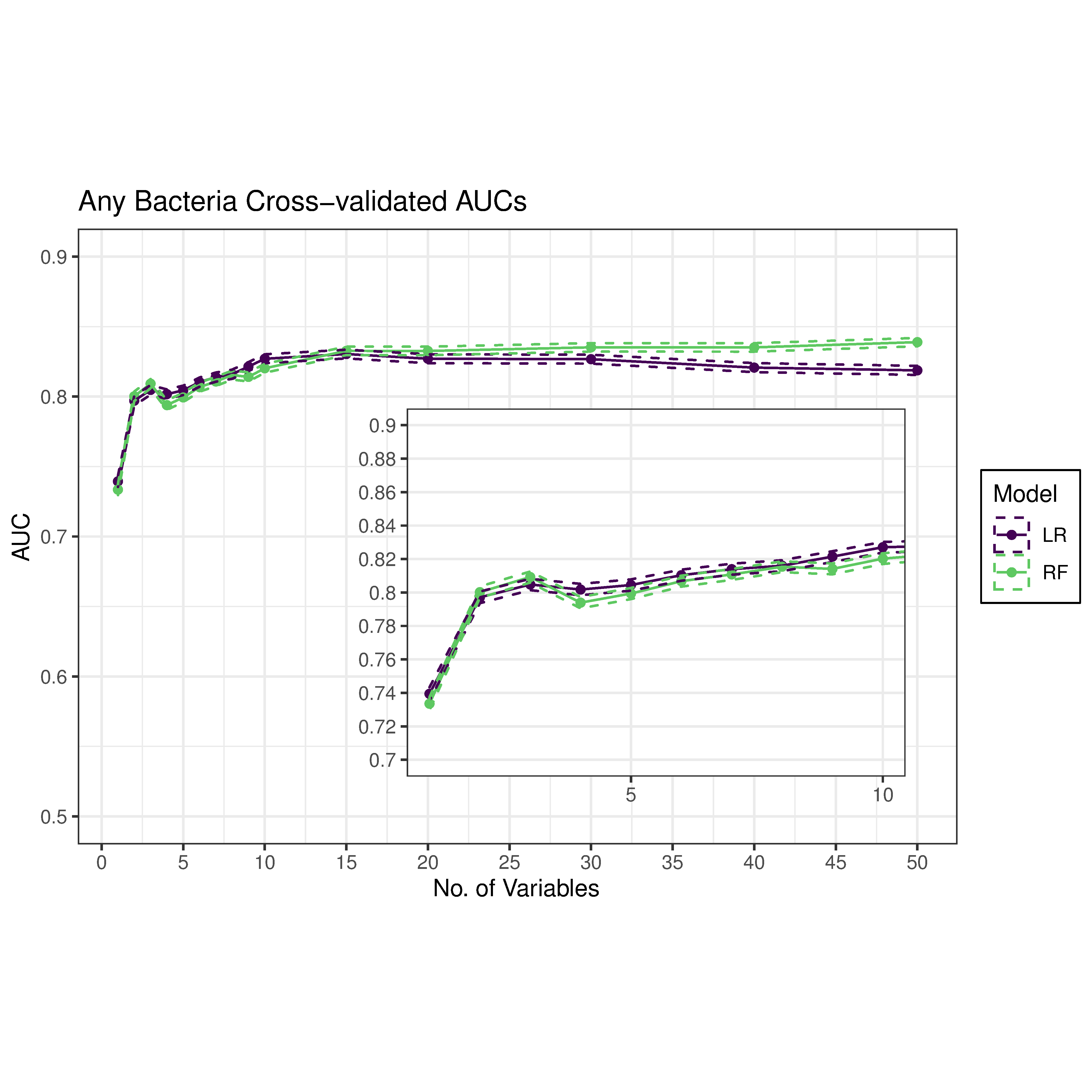

Supplement: S3 Fig — Note that we only filtered out non-responses for response variables that were in the top 50 of our screening step. (TIF) [file pntd.0008677.s005.tif]

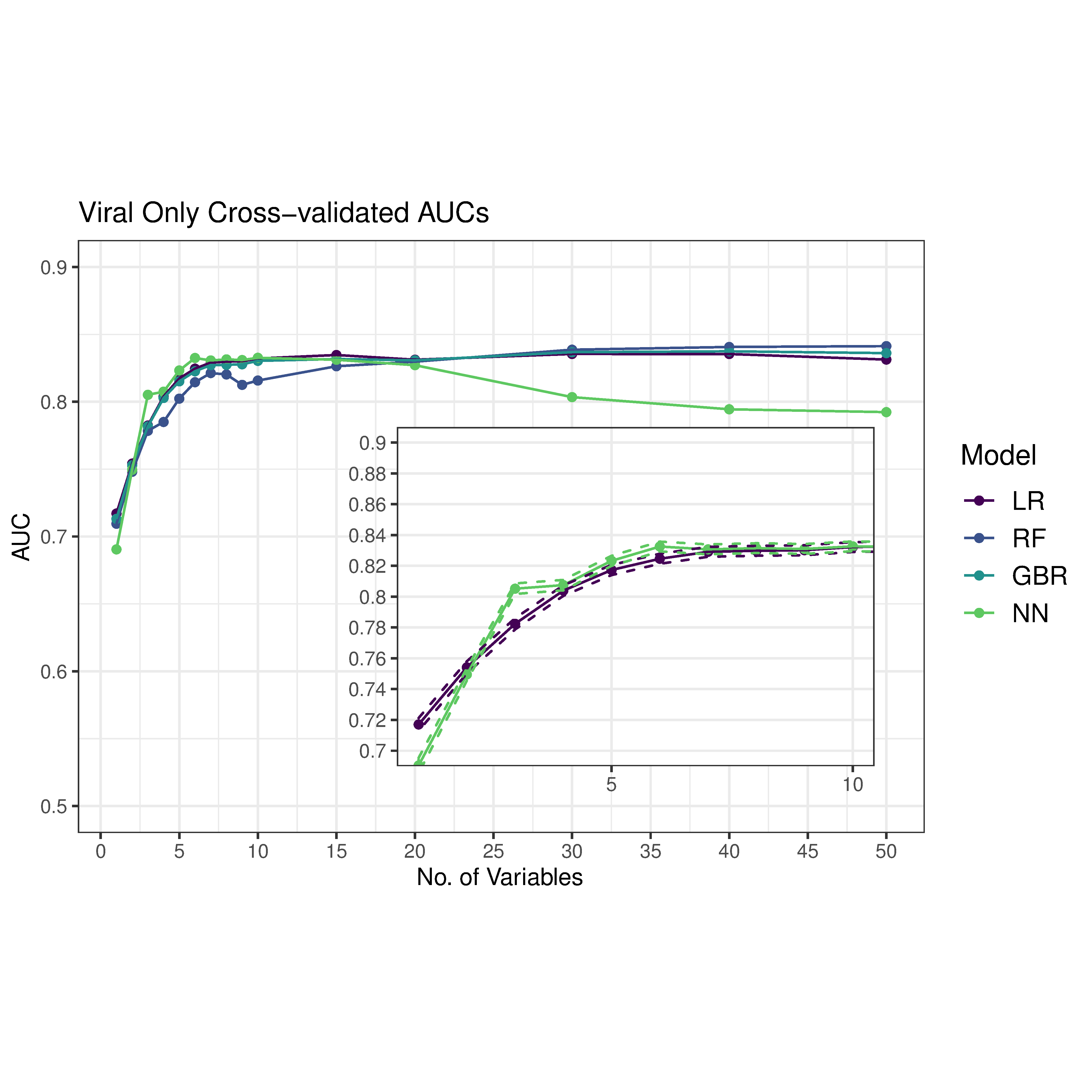

Supplement: S4 Fig — (TIF) [file pntd.0008677.s006.tif]

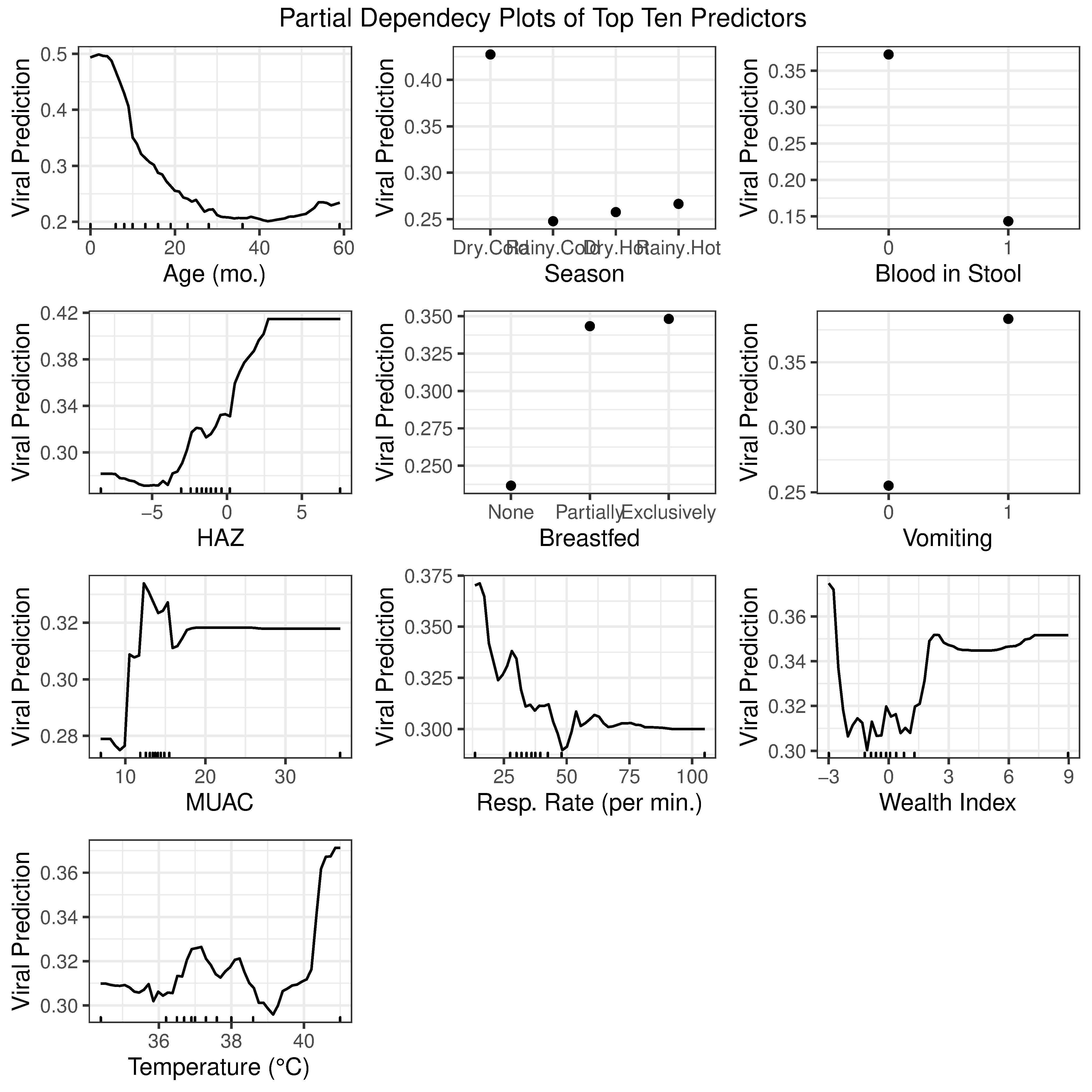

Supplement: S5 Fig — Ticks on the x-axis show the deciles of the data. (TIF) [file pntd.0008677.s007.tif]

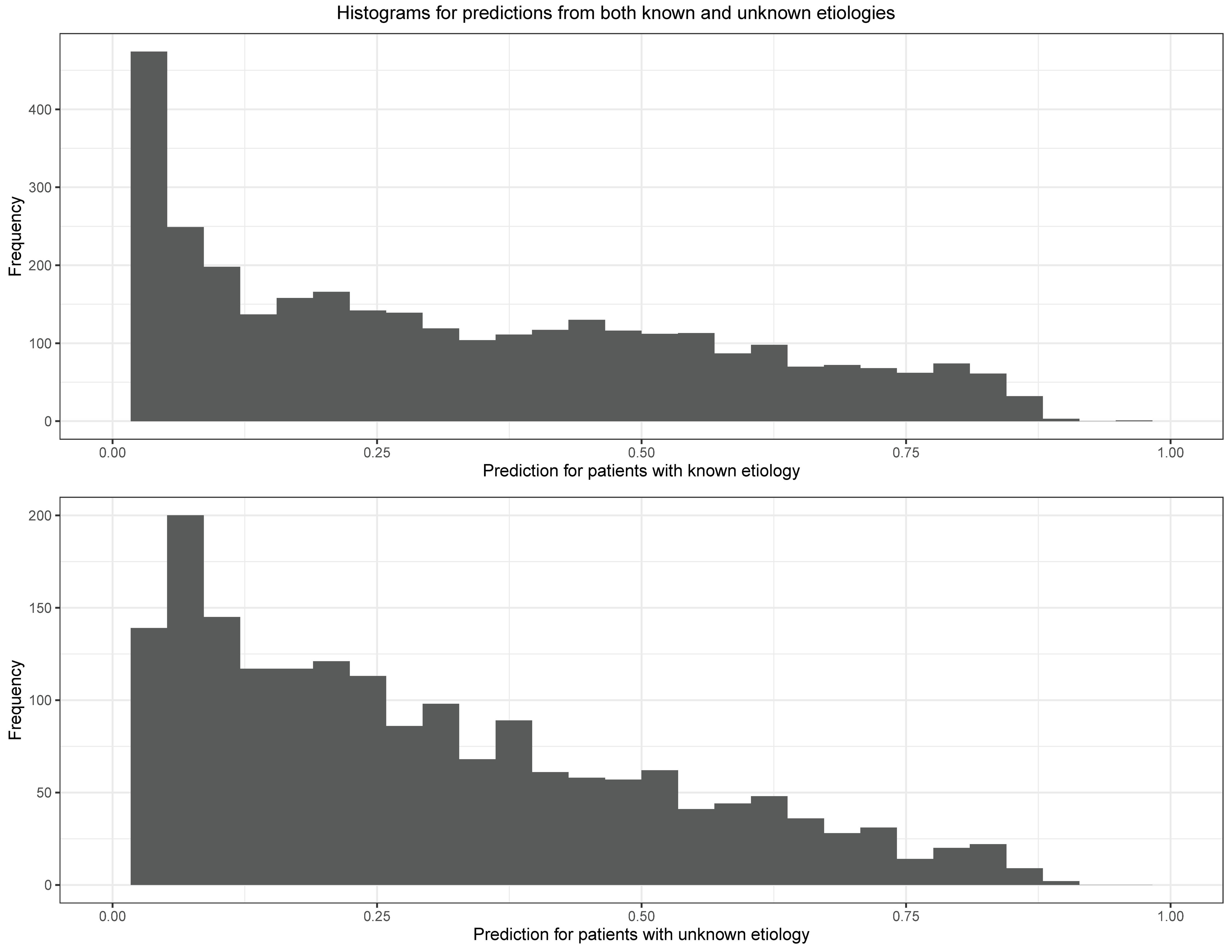

Supplement: S6 Fig — (TIF) [file pntd.0008677.s008.tif]

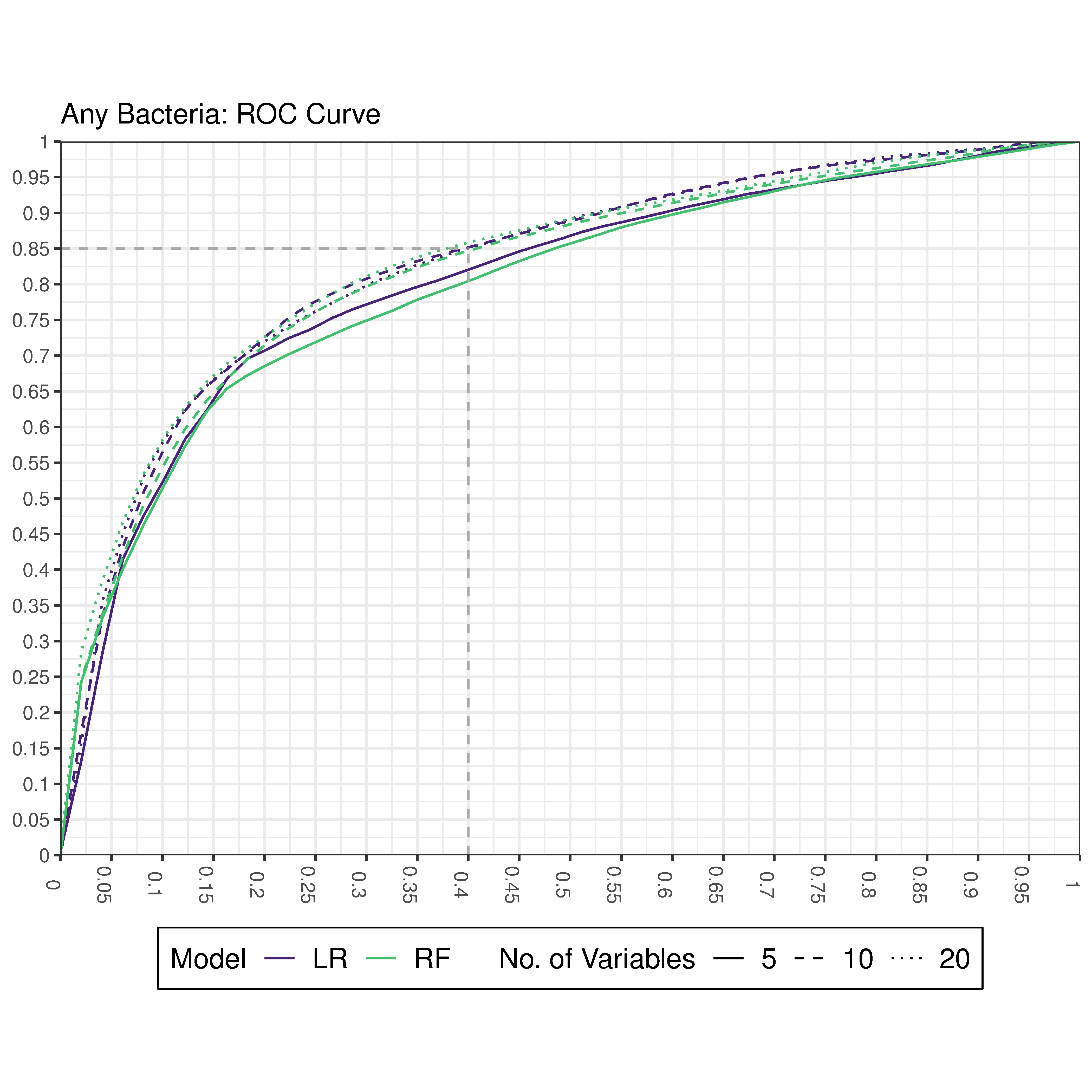

Supplement: S7 Fig — The faded dashed lines represent examples of how we could achieve a sensitivity of 0.85 and a specificity of 0.59 for any bacteria. (TIF) [file pntd.0008677.s009.tif]
