# Supplementary material for: Clinical predictors for etiology of acute diarrhea in children in resource-limited settings
Source: PLoS Negl Trop Dis. 2020 Oct 9;14(10):e0008677. doi: 10.1371/journal.pntd.0008677 (PMC7588112; doi:10.1371/journal.pntd.0008677)
Supplement: S1 Table — (DOCX) [file pntd.0008677.s010.docx]

S1 Table: Rank of variable importance for predicting bacterial etiology by reduction in residual sum of squares (RSS) using random forest regression.

| Variable Name | RSS Reduction |
| --- | --- |
| Age | 73.9 |
| Blood in stool | 33.9 |
| Breastfed | 29.1 |
| HAZ | 25.9 |
| MUAC | 24.3 |
| Resp. Rate | 20.6 |
| Season | 19.9 |
| Wealth Index | 19.7 |
| Temperature | 18.9 |
| Vomiting | 18 |
