# Supplementary material for: Clinical predictors for etiology of acute diarrhea in children in resource-limited settings
Source: PLoS Negl Trop Dis. 2020 Oct 9;14(10):e0008677. doi: 10.1371/journal.pntd.0008677 (PMC7588112; doi:10.1371/journal.pntd.0008677)
Supplement: S2 Table — (DOCX) [file pntd.0008677.s011.docx]

S2 Table: The table contains all predictors considered by the random forest variable screening as well as the relative order of importance (reduction in mean square error (MSE) by the variable’s inclusion) for viral prediction for the whole dataset.

| Variable | Reduction MSE | Variable | Reduction MSE | Variable | Reduction MSE | Variable | Reduction MSE | Variable | Reduction MSE | Variable | Reduction MSE |
| --- | --- | --- | --- | --- | --- | --- | --- | --- | --- | --- | --- |
| base_age | 51.391 | f4a_fac_waste | 5.143 | f4a_drh_lethrgy | 3.083 | f4a_water_pubtap | 2.342 | f4a_drh_breath | 1.448 | ani_horse | 0.813 |
| Season | 28.678 | f4a_drh_strain | 5.113 | f4a_wash_cook | 3.081 | f4a_fetch_water | 2.147 | f4a_seek_doc | 1.402 | f4b_abn_hair | 0.793 |
| f4a_drh_blood | 26.351 | f4b_mouth | 4.77 | f4b_skin | 3.057 | f4a_store_water | 2.142 | f4a_house_cart | 1.395 | f4a_seek_other | 0.728 |
| f4b_haz | 24.746 | f4a_max_stools | 4.764 | f4a_cur_drymouth | 3.031 | f4a_house_scoot | 2.141 | f4a_wash_othr | 1.395 | ani_pig | 0.725 |
| f4a_drh_vomit | 22.741 | f4b_recommend | 4.734 | f4a_ani_rodents | 3.009 | f4a_hometrt_othr1 | 2.1 | f4a_fuel_crop | 1.331 | f4a_fuel_other | 0.637 |
| f4a_breastfed | 21.422 | f4b_mental | 4.604 | f4a_wash_use | 3.001 | f4a_house_agland | 2.058 | f4a_fuel_coal | 1.33 | f4a_water_covwell | 0.585 |
| f4b_muac | 21.241 | f4a_floor | 4.425 | f4a_wash_def | 2.958 | f4a_house_elec | 2.054 | f4a_water_house | 1.191 | f4a_water_pond | 0.529 |
| f4b_resp | 18.652 | f4a_drh_thirst | 4.329 | f4a_trt_water | 2.925 | f4a_notrt_water | 2.016 | f4a_hometrt_othr2 | 1.18 | f4a_drh_conv | 0.525 |
| wealth index scale | 18.633 | f4a_dad_live | 4.155 | f4a_house_bike | 2.884 | f4a_hometrt_zinc | 1.985 | f4a_water_rain | 1.174 | f4a_water_well | 0.512 |
| f4b_temp | 17.083 | f4a_water_avail | 4.048 | f4a_hometrt_none | 2.862 | f4a_fuel_charcoal | 1.981 | f4a_house_car | 1.138 | f4a_water_covpwell | 0.51 |
| f4a_ppl_house | 14.015 | f4a_trip_week | 4.034 | f4a_cur_restless | 2.762 | f4a_ani_no | 1.945 | f4a_water_pubwell | 1.134 | f4a_mom_live | 0.456 |
| f4a_share_fac | 10.489 | f4a_ani_cat | 3.885 | f4a_seek_outside | 2.72 | f4a_hometrt_ab | 1.93 | ani_donkey | 1.11 | f4a_seek_friend | 0.456 |
| f4b_nature_stool | 9.985 | f4a_cur_thirsty | 3.798 | f4a_ani_fowl | 2.718 | f4a_ani_sheep | 1.919 | f4b_skin_pinch | 1.099 | f4a_hometrt_othrliq | 0.447 |
| f4a_drh_days | 9.984 | f4a_fuel_kero | 3.655 | f4a_drh_restless | 2.695 | f4a_cur_fastbreath | 1.912 | f4a_water_river | 1.09 | f4a_water_othr | 0.425 |
| f4a_slp_rooms | 9.151 | f4b_under_nutr | 3.635 | f4a_house_tele | 2.659 | f4a_seek_pharm | 1.805 | f4a_primcare | 1.089 | f4a_house_boat | 0.373 |
| f4a_trip_day | 9.1 | f4b_admit | 3.608 | f4a_ani_cow | 2.639 | f4a_relationship | 1.786 | f4a_water_shallwell | 1.078 | f4a_ani_other | 0.328 |
| f4a_ms_water | 8.284 | f9_memory_aid | 3.544 | f4a_cur_lethrgy | 2.614 | f4a_fuel_natgas | 1.761 | f4a_water_bought | 1.056 | f4b_skin_flaky | 0.312 |
| f4a_yng_children | 7.997 | f4a_drh_cough | 3.454 | f4a_ani_goat | 2.577 | f4a_house_fridge | 1.722 | f4a_drh_undrink | 1.036 | f4a_water_unspring | 0.278 |
| Site | 7.766 | f4a_wash_nurse | 3.344 | f4a_drh_lessdrink | 2.519 | f4a_fuel_grass | 1.702 | f4a_fuel_dung | 1.025 | f4a_drh_prolapse | 0.277 |
| f4a_prim_schl | 7.421 | f4a_cur_skin | 3.342 | f3_drh_turgor | 2.517 | f4a_water_yard | 1.681 | f4a_seek_healer | 0.992 | f4a_water_prospring | 0.261 |
| wealth index cat. | 7.187 | f4a_ani_dog | 3.262 | f4a_fuel_wood | 2.517 | f4a_fuel_propane | 1.64 | f4b_chest_indrw | 0.98 | f4a_hometrt_milk | 0.215 |
| f4a_disp_feces | 6.794 | f3_gender | 3.262 | f4a_house_phone | 2.506 | f4a_hometrt_herb | 1.635 | f4a_seek_remdy | 0.884 | f4b_bipedal | 0.205 |
| f4b_eyes | 6.756 | f3_drh_iv | 3.258 | f4a_wash_eat | 2.487 | f4a_wash_animal | 1.599 | f4a_drh_consc | 0.872 | f4a_fuel_elec | 0.144 |
| f4a_offr_drink | 6.072 | f4a_wash_child | 3.157 | f3_drh_hosp | 2.421 | f4a_chlorine | 1.596 | f4a_fuel_biogas | 0.868 | f4b_rectal | 0.11 |
| f4a_drh_bellypain | 5.226 | f4a_drh_fever | 3.108 | f4a_house_radio | 2.397 | f4a_water_deepwell | 1.552 | f4a_water_bore | 0.828 | f4b_observe_stool | 0.052 |
| f4a_trt_method | 5.211 | f4a_hometrt_ors | 3.107 | f4a_hometrt_maize | 2.374 | f4a_seek_privdoc | 1.492 | f4a_house_none | 0.816 | f4b_volume | 0.012 |
