# Supplementary material for: Clinical predictors for etiology of acute diarrhea in children in resource-limited settings
Source: PLoS Negl Trop Dis. 2020 Oct 9;14(10):e0008677. doi: 10.1371/journal.pntd.0008677 (PMC7588112; doi:10.1371/journal.pntd.0008677)
Supplement: S3 Table — (DOCX) [file pntd.0008677.s012.docx]

S3 Table: Steyerberg’s A and B (intercept and slope) for both logistic regression and random forest regression models fit with a different number of variables

| **Method** | **Logistic Regression** | | **Method** | **Random Forest** | |
| --- | --- | --- | --- | --- | --- |
| **# of Vars.** | **Intercept (A)** | **Slope (B)** | **# of Vars.** | **Intercept (A)** | **Slope (B)** |
| 1 | -0.0207 | 1.06 | 1 | 0.00665 | 0.974 |
| 2 | -0.00154 | 0.994 | 2 | 0.0211 | 0.922 |
| 3 | 0.00796 | 0.976 | 3 | 0.0286 | 0.911 |
| 4 | 0.00666 | 0.97 | 4 | 0.0508 | 0.83 |
| 5 | 0.00584 | 0.981 | 5 | 0.0375 | 0.88 |
| 6 | 0.00542 | 0.972 | 6 | 0.035 | 0.878 |
| 7 | 0.00224 | 0.985 | 7 | 0.0279 | 0.905 |
| 8 | 0.00452 | 0.978 | 8 | 0.0315 | 0.893 |
| 9 | 0.00582 | 0.977 | 9 | 0.0472 | 0.847 |
| 10 | 0.00692 | 0.976 | 10 | 0.0454 | 0.854 |
| 15 | 0.00783 | 0.968 | 15 | 0.0365 | 0.875 |
| 20 | 0.0173 | 0.949 | 20 | 0.0441 | 0.864 |
| 30 | 0.0239 | 0.924 | 30 | 0.0522 | 0.833 |
| 40 | 0.0276 | 0.914 | 40 | 0.054 | 0.83 |
| 50 | 0.0345 | 0.883 | 50 | 0.0562 | 0.814 |
