# Supplementary material for: Clinical predictors for etiology of acute diarrhea in children in resource-limited settings
Source: PLoS Negl Trop Dis. 2020 Oct 9;14(10):e0008677. doi: 10.1371/journal.pntd.0008677 (PMC7588112; doi:10.1371/journal.pntd.0008677)
Supplement: S4 Table — (DOCX) [file pntd.0008677.s013.docx]

S4 Table: The odds ratios, 95% confidence interval, and p-value from a logistic regression model for the viral only outcome for the top 5 variables.

| Variable Name | Odds Ratios (95% CI) | P-value |
| --- | --- | --- |
| Intercept | 3.046 (2.400 – 3.865) | <0.0001 |
| Age (mo.) | 0.945 (0.937 – 0.954) | <0.0001 |
| Season |  |  |
| Dry/Cold | Reference |  |
| Rainy/Cold | 0.220 (0.165 – 0.294) | <0.0001 |
| Dry/Hot | 0.290 (0.233 – 0.362) | <0.0001 |
| Rainy/Hot | 0.356 (0.284 – 0.446) | <0.0001 |
| Blood in stool | 0.141 (0.105 – 0.188) | <0.0001 |
| HAZ | 1.220 (1.142 – 1.305) | <0.0001 |
| Vomiting | 2.391 (2.007 – 2.849) | <0.0001 |
