# Supplementary material for: Clinical predictors for etiology of acute diarrhea in children in resource-limited settings
Source: PLoS Negl Trop Dis. 2020 Oct 9;14(10):e0008677. doi: 10.1371/journal.pntd.0008677 (PMC7588112; doi:10.1371/journal.pntd.0008677)
Supplement: S5 Table — (DOCX) [file pntd.0008677.s014.docx]

S5 Table: The estimate, 95% confidence interval, and p-value from a logistic regression model for the any bacteria outcome.

| Variable | Beta Estimate (95%CI) | P-value |
| --- | --- | --- |
| Intercept | -1.9330 (-5.3748 - 1.5089) | 0.271 |
| Age (mo.) | 0.0567 (0.0455 - 0.0679) | <0.0001 |
| Blood in stool | 2.0062 (1.7533 - 2.2591) | <0.0001 |
| Breastfed |  |  |
| None | Reference |  |
| Partially | -0.7296 (-0.9592 - -0.4999) | <0.0001 |
| Exclusively | -0.6245 (-1.0411 - -0.2078) | 0.0033 |
| HAZ | -0.1036 (-0.1768 - -0.0305) | 0.0055 |
| MUAC | -0.0353 (-0.1029 - 0.0322) | 0.305 |
| Resp. Rate (per min.) | 0.0131 (0.0028 - 0.0234) | 0.0124 |
| Season |  |  |
| Dry/Cold | Reference |  |
| Rainy/Cold | 0.9156 (0.6344 - 1.1969) | <0.0001 |
| Dry/Hot | 0.9725 (0.7595 - 1.1855) | <0.0001 |
| Rainy/Hot | 0.8389 (0.6164 - 1.0614) | <0.0001 |
| Wealth Index | -0.0211 (-0.1053 - 0.0631) | 0.623 |
| Temperature (◦C) | 0.0299 (-0.0621 - 0.1218) | 0.5241 |
| Vomiting | -0.6766 (-0.846 - -0.5071) | <0.0001 |
