# Supplementary material for: Clinical predictors for etiology of acute diarrhea in children in resource-limited settings
Source: PLoS Negl Trop Dis. 2020 Oct 9;14(10):e0008677. doi: 10.1371/journal.pntd.0008677 (PMC7588112; doi:10.1371/journal.pntd.0008677)
Supplement: S6 Table — (DOCX) [file pntd.0008677.s015.docx]

S6 Table: The estimate, 95% confidence interval, and p-value from a logistic regression model for the viral only outcome with rotavirus cases removed.

| Variable Name | Odds Ratios (95% CI) | P-value |
| --- | --- | --- |
| Intercept | 1.693 (0.006 – 488.504) | 0.855 |
| Age (mo.) | 0.951 (0.933 – 0.969) | <0.0001 |
| Season |  |  |
| Dry/Cold | Reference |  |
| Rainy/Cold | 0.390 (0.246 – 0.617) | <0.0001 |
| Dry/Hot | 0.550 (0.385 – 0.786) | 0.001 |
| Rainy/Hot | 0.600 (0.415 – 0.866) | 0.006 |
| Blood in stool | 0.154 (0.096 – 0.248) | <0.0001 |
| HAZ | 1.085 (0.964 – 1.220) | 0.176 |
| Vomiting | 1.578 (1.194 – 2..85) | 0.001 |
| Breastfed |  |  |
| None | Reference |  |
| Partially | 2.281 (1.520 – 3.424) | <0.0001 |
| Exclusively | 1.827 (0.921 – 3.622) | 0.085 |
| MUAC | 1.061 (0.965 – 1.167) | 0.220 |
| Resp. Rate (per min.) | 0.982 (0.965 – 1.000) | 0.047 |
| Wealth Index | 1.099 (0.958 – 1.260) | 0.178 |
| Temperature (◦C) | 0.954 (0.819 – 1.112) | 0.550 |
