# Supplementary material for: Clinical predictors for etiology of acute diarrhea in children in resource-limited settings
Source: PLoS Negl Trop Dis. 2020 Oct 9;14(10):e0008677. doi: 10.1371/journal.pntd.0008677 (PMC7588112; doi:10.1371/journal.pntd.0008677)
Supplement: S7 Table — Lastly, it shows the AUC and a 95% confidence interval resulting from testing the logistic regression with variables based on the overall variable importance on each site individually following its training on the other countries in the same continent. (DOCX) [file pntd.0008677.s016.docx]

S7 Table: The table contains both site-specific variable importance ordering and a cross-validated average overall AUC, AUC by country, and AUC by continent and confidence intervals from a 5 (bold) and 10 (ital.) variable logistic regression model for predicting a viral etiology with variables based on the overall variable importance. Lastly, it shows the AUC and a 95% confidence interval resulting from testing the logistic regression with variables based on the overall variable importance on each site individually following its training on the other countries in the same continent.

9

|  | Africa | | | | Asia | | |
| --- | --- | --- | --- | --- | --- | --- | --- |
| Variable/Country | The Gambia | Mali | Mozambique | Kenya | India | Bangladesh | Pakistan |
| 1 | Age | Age | Age | Age | Age | Age | Age |
| 2 | Blood in stool | MUAC | Breastfed | Breastfed | MUAC | Blood in stool | MUAC |
| 3 | HAZ | Breastfed | HAZ | HAZ | HAZ | Eyes | HAZ |
| 4 | Breastfed | HAZ | MUAC | MUAC | Season | Vomiting | Breastfed |
| 5 | MUAC | Resp. Rate | Season | Resp. Rate | Resp. Rate | Season | Resp. Rate |
| 6 | Temp. | Wealth Index | Resp. Rate | Temp. | Blood in stool | MUAC | Wealth Index |
| 7 | Resp. Rate | Temp. | Temp. | Wealth Index | Wealth Index | Rectal Straining | Temp. |
| 8 | Season | Ppl. of House | Wealth Index | Ppl. of House | # Share Fac. | Temp. | Ppl. of House |
| 9 | Wealth Index | Vomiting | Vomiting | # Share Fac. | Temp. | HAZ | Blood in stool |
| 10 | Ppl. of House | Season | Blood in stool | Water Source | Ppl. of House | Wealth Index | Days of Episode |
| Country AUCs | **0.827 (0.818-0.836)** | **0.741 (0.731-0.752)** | **0.802 (0.791-0.813)** | **0.763 (0.752-0.773)** | **0.775 (0.767-0.784)** | **0.892 (0.885-0.898)** | **0.760 (0.750-0.770)** |
|  | 0.840 (0.832-0.849) | 0.756 (0.746-0.767) | 0.831 (0.821-0.841) | 0.773 (0.763-0.784) | 0.807 (0.799-0.815) | 0.921 (0.915-0.927) | 0.765 (0.755-0.775) |
| Continent AUCs | **0.779 (0.774-0.785)** | | | | **0.826 (0.821-0.830)** | | |
|  | 0.797 (0.792-0.802) | | | | 0.850 (0.845-0.854) | | |
| Overall AUC | **0.806 (0.803-0.809)** | | | | | | |
|  | 0.829 (0.825-0.832) | | | | | | |
| Continent External Validation | **0.746 (0.696-0.797)** | **0.711 (0.657-0.765)** | **0.773 (0.722-0.823)** | **0.723 (0.672-0.773)** | **0.772 (0.735-0.810)** | **0.897 (0.868-0.926)** | **0.765 (0.719-0.811)** |
|  | 0.767 (0.719-0.815) | 0.790 (0.736-0.842) | 0.831 (0.787-0.876) | 0.715 (0.664-0.766) | 0.811 (0.777-0.846) | 0.930 (0.906-0.953) | 0.782 (0.738-0.826) |
